# Voluntary attendance of small-group brainstorming tutoring courses intensify new clerk’s “excellence in clinical care”: a pilot study

**DOI:** 10.1186/s12909-016-0843-6

**Published:** 2017-01-06

**Authors:** Ling-Yu Yang, Chia-Chang Huang, Hui-Chi Hsu, Ying-Ying Yang, Ching-Chi Chang, Chiao-Lin Chuang, Wei-Shin Lee, Jen-Feng Liang, Hao Min Cheng, Chin-Chou Huang, Fa-Yauh Lee, Shung-Tai Ho, Ralph Kirby

**Affiliations:** 1 Division of Clinical Skills Training, Yilan, Taiwan; 2 Department of Medical Education, Yilan, Taiwan; 3 Department of Medicine, Division of General Medicine, Yilan, Taiwan; 4 Taipei Veterans General Hospital, Yilan, Taiwan; 5 Camillians Saint Mary’s Hospital Luodong, Yilan, Taiwan; 6 Department of Medicine, National Yang-Ming University, Taipei, Taiwan

**Keywords:** Clerkship, Clinical excellence, Diagnostic and clinical reasoning skills, Small-group brainstorming, Tutoring courses

## Abstract

**Background:**

Clerkship provides a unique way of transferring the knowledge and skills gathered during medical school’s curriculum into real-ward clinical care environment. The annual program evaluation has indicated that the training of clerks in diagnostic and clinical reasoning skills needed to be enhanced. Recently, “clinical excellence” program have been promoted in our institution to augment the excellence in clinical care of new clerks. Current study aims to evaluate whether this pilot program improve the “clinical excellence” of new clerks.

**Methods:**

In a pilot study, groups of new clerks in years 2013 and 2014 voluntarily attended either a small-group brainstorming course or a didactic classroom tutoring courses as part of their 3-month internal medicine clinical rotation block. A third group of new clerks did not join either of the above courses and this group served as the control group. Pre-block/post-block self-assessment and post-block 5-station mini-Objective Subjective Clinical Examinations (OSCEs) were used to evaluate the effectiveness of these two additional courses that trained diagnostic and clinical reasoning skills.

**Results:**

Overtime, the percentages of new clerks that attended voluntarily either the small-group brainstorming or classroom tutoring courses were increased. Higher post-block self-assessed diagnostic and clinical reasoning skill scores were found among individuals who attended the small-group brainstorming courses compared to either the didactic group or the control group. In a corresponding manner, the small-group brainstorming group obtained higher summary OSCE_diag_ and OSCE_reason_ scores than either the didactic group or control group. For all basic images/laboratory OSCE stations, the individual diagnostic skill (OSCE_diag_) scores of the small-group brainstorming group were higher than those of the didactic group. By way of contrast, only the clinical reasoning skill (OSCE_reason_) scores of the basic electrocardiogram and complete blood count + biochemistry OSCE station of thesmall-group brainstorming group were higher than those of the didactic group. Among the small-group brainstorming group, clerks with higher cumulative learning hours (>30-h) had significant higher OSCE_diag_ and OSCE_reason_ scores (>400) than those with less cumulative learning hours.

**Conclusion:**

Our pilot study provides a successful example of the use of a small-group tutoring courses for augmenting the diagnostic and clinical reasoning skills of new clerks. The positive results obtained during the initial 2-year long pilot “clinical excellence” program have encouraged the formal implementation of this course as part of the clerkship curriculum.

**Electronic supplementary material:**

The online version of this article (doi:10.1186/s12909-016-0843-6) contains supplementary material, which is available to authorized users.

## Background

Most program directors reported that the clinical care competencies of clerks are not adequately trained before starting core clinical clerkship [1]. It is suggested that medical educator cannot rely on clerkship experiences alone to provide adequate basic clinical skills training [2]. Additional skills training during clinical rotation seems to offer the students a superior preparation for clerkships as well as influencing the students’ learning abilities during the clerkships [3]. This is supported by the better OSCE performance of clerks who have received extra-skill training compared to those who have received traditional bedside teaching alone [4, 5]. It is suggested that the implementation of well-constructed clinical training course, especially diagnostic and clinical reasoning skills, is a highly effective way of preparing clerks for real-ward clinical care [2, 6].

A growing number of medical educators have suggested that active team learning sessions should be paralleled by didactic classroom lecture-based teaching sessions. Notwithstanding the above, the clerkship curriculum is already very condensed and fully-occupies the participant in our system. As a result, it has become necessary to assess the effects of such an extra-curriculum course before it is permanently introduced into the regular clerkship course. As a preliminary step, our clerkship committee leads a “clinical excellence” program that constructed two additional small-group brainstorming course and a classroom didactic course. These two courses are designed to improve the diagnostic and clinical reasoning skills of new clerks. Specifically, these courses’ contents emphasized the ability to assemble knowledge rather than memorize knowledge.

Accordingly, the aim of this study was to determine whether voluntary participation in either the small-group brainstorming course or classroom didactic course effectively enhanced the excellence in clinical care of new clerks with respect to diagnostic and clinical reasoning skills.

## Method

Taipei Veteran General Hospital (Taipei VGH) is a medical center that is certificated as the formal teaching hospital of National Yang-Ming medical university. The curriculum at National Yang-Ming medical university and Taipei VGH consists of 7 years of teaching; which consists of, 2 years of university teaching (first and second years), 2 years of preclinical training (third and fourth years) and 3 years of preclinical training (clerkship and internship), these are followed by 1 year of postgraduate residency and several years of sub-specilaity residency training.

### Background for implementation of the pilot clerkship “clinical excellence” program

The annual end-of-clerkship web-based surveyed (Additional file 1: Table S1) from the class 2012 clerks suggested that additional diagnostic and clinical reasoning skills tutoring courses were needed in order to promote excellence in clinical care. After combining this annual report with information from the literature [5–10], the opinion of current internship trainee’s and the performance data from the previous 3-month internal medicine block OSCEs, there were five diagnostic and clinical reasoning skills that seemed to need augmentation and these are listed in Tables 1 and 2.

**Table 1:** Content of clerkship tutoring course in internal medicine block in the “clinical excellence” program

1. Basic electrocardiogram (EKG) diagnostic and clinical reasoning skills
2. Basic chest X-ray (CXR) diagnostic and clinical reasoning skills
3. Basic abdominal plain film (KUB) diagnostic and clinical reasoning skills
4. Basic arterial blood gas (ABG) diagnostic and clinical reasoning skills
5. Basic complete blood count (CBC) and biochemistry diagnostic and clinical reasoning skills

**Table 2:** Detail content of 5 post-Internal Medicine block OSCE of all class 2013 and class 2014 clerks

	Clinical scenario that SP present to the examinee	Mission of examinee
1^st^ station	A regular hemodialysis uremia patient who had suffered from 2-day of palpitation symptoms after 3 days of diarrhea. SP will presented with a second degree Mobitz type II AV block EKG after a brief self-introduction	interpret the EKG (diagnostic skills), undertake a focused history taking, explain the appropriate differential diagnosis and describe a further treatment plan (clinical reasoning skill) to the SP
2^nd^ station	A productive cough patient with fever and the SP presented with a CXR showing a pneumonia patch	interpret the CXR (diagnostic skills), undertake a focused history taking, explain the appropriate differential diagnosis and describe a further treatment plan (clinical reasoning skill) to the SP
3^rd^ station	A patient with abdominal pain due to bowel obstruction and the SP presents with an intestinal obstruction KUB.	interpret the KUB (diagnostic skills), undertake a focused history taking, explain the appropriate differential diagnosis and describe a further treatment plan (clinical reasoning skill) to the SP
4^th^ station	A patient shows confusion and dyspnea with ABG showed metabolic acidosis is presented by SP	interpret the CBC + biochemistry (diagnostic skills), undertake a focused history taking, explain the appropriate differential diagnosis and describe a further treatment plan (clinical reasoning skill) to the SP
5^th^ station	SP is a case of acute alcoholic hepatitis with jaundice and leukocytosis	interpret the ABG (diagnostic skills), undertake a focused history taking, explain the appropriate differential diagnosis and describe a further treatment plan (clinical reasoning skill) to the SP

### Description of the clerkship “clinical excellence” program

#### Participants

Overall, this additional “clinical excellence” program had the aim of providing both a small-group brainstorming course and a didactic classroom tutoring courses; these were held parallel. All clerks from the classes of 2013 and 2014 could voluntarily join one of the two groups during their 3-month internal medicine block. Additionally, there was a third group of clerks who did not join either course; these individuals formed the control group and were provided a manual that included handouts and relevant references; these targeted the diagnostic and clinical reasoning skills trained by the two courses and allowed self-directed study. To avoid any unnecessary interference with their essential training, two additional tutoring courses were held after work and at weekend. Clerks not participating in this study had no disadvantages regarding the passing/failing the internal medicine clerkship block or end-of block OSCE.

Since the day entrance their 3-month Internal Medicine block, all clerks were divided into three groups [small-group tutoring, classroom lecture tutoring and regular control] according to their choice. Before the beginning of Internal Medicine clerkship, every clerk draws lots to decide their combination of the subspecialties with 3-month block. After the initial pre-block self assessment, all clerks simultaneously receive the post-Internal Medicine block OSCE and self assessment at the end of block.

#### Small group brainstorming course

This regular 2-week course was provided by internal medicine clerkship committee. Specifically, the course covered EKG, CXR, KUB, ABG, and CBC/biochemistry diagnostic and clinical reasoning skills’ training sessions. With group sizes ranging from 15 to 20 clerks, there were five regular faculty tutors who were responsible for developing and administrating the small-group brainstorming courses. Each diagnostic and clinical reasoning skills-enhancement session was piloted and reviewed by a committee prior to introduction into the schedule.

Each session lasted for two and a half hours. During the first 30-min, the participants were given basic clinical information related to one of five types of data by a teaching assistant; these sets were EKG, CXR, KUB, ABG or CBC+ biochemistry findings. The CXRs and KUBs datasets were tutored by radiology faculty members, the ABGs and the CBC + biochemistry datasets were tutored by generalist faculty members and the EKGs datasets were tutored by cardiology faculty members. After an initial 1 hour of small-group discussion and internet searching, specific course-developed faculty tutoring took place during the second hours of each course. As part of the training in diagnostic and clinical reasoning skills, each participant needed to present a lettered card corresponding to their final answer, namely what they believed to be the further assessment, diagnosis or treatment needed based on the real patient-based EKG, CXR, KUB, ABG or CBC + biochemistry datasets. The multiple-choice format of the problems allowed the tutor and participants to have instantaneous feedback regarding each participant’s answers. Before providing the final correct answers, the tutor then led a discussion in which the participants defended and debated their answers.

#### The didactic classroom tutoring course

The internal medicine clerkship committee provided an equivalent amount of regular 2-week classroom lectures for the clerks whose did not attend the above small-group brainstorming course. Similarly to the above small-group brainstorming course, each lecture also consisted of two and a half hours; these involved another five regular faculties that receiving similar training and preparation as those in small-group brainstorming course. The same well-constructed content and goals (training in diagnostic and clinical reasoning skills) as small-group brainstorming course were included within these classroom tutoring lectures. Without any limit to the participant number (18–24 participants on average), these five sessions were scheduled once every 2 weeks and repeated six times over the 3-month internal medicine block.

### Schedules of training session in small-group and classroom tutoring courses

Specific sessions in regular 2-week small-group and classroom tutoring courses were scheduled once every 2 weeks and repeated for 6 times within the 3-month internal medicine block. Thus, clerks were free to book their attendance in advance. Monthly changes in the teaching material increase the participants’ exposure to a variety of clinical situations during each category of small-group and classroom tutoring courses. In other words, the contents of training in either small-group or classroom lecture courses were similar in the first 2-week and second 2-week of each month but different among first, second and third month within each 3-month intervals. Clerks were asked to choose either first 2-week or second 2-week small-group or classroom tutoring courses in every month of their Internal-Medicine blocks. Specifically, their attendance should cover all 3 months, with different regular changed teaching materials, in their 3-month Internal Medicine block. Each voluntary participant needed to complete a required 25-h [(2.5*5 = 12.5 h/week)*2 = 25-h] of small-group brainstorming or classroom lecture courses during their 3-month Internal-Medicine block.

In addition to minimal required 25-h of this program, participants were encouraged to attend these sessions as frequent as possible in order to further enhance their “excellence in clinical care”. In participants that already reach minimal required hours, additional courses can be participated freely as 0.5-h, 1-h, 1.5-h, 2-h or 2.5-h dependent on the availability of their schedule. In other words, additional courses participants can either solely observation or actively join the courses and teaching assistance will check their joined hour every half hour. Finally, the relationship between teaching assistant-calculated total exposure hours of each participant and the participant’s post-Internal-Medicine block “clinical excellence” OSCE performance were analyzed. Usually, these classroom tutoring lectures were held parallel and at the same time as the small-group brainstorming courses after work and during weekends.

### The pre- and post- internal medicine block self-assessment

In order to check whether the basal degrees of “clinical excellence” were different among clerks that with different combined 6-sub-specialities of clinical rotation in their 3-month Internal Medicine block. The pre-Internal Medicine block self assessments for “clinical excellence” were completed by all class 2013 and class 2014 clerks.

All clerks (fifth-year medical students, the classes of 2013 and 2014), whether or not they participated in the small-group brainstorming course, the didactic classroom course or neither, were asked to complete an web-delivered pre-block and post-block self-assessment for their degree of excellence in clinical care, which called self-assessed “clinical excellence” survey (Additional file 2: Table S2) to allow comparison between the three groups. The post-block self-assessment was completed before the post-Internal-Medicine block OSCE. In particular, the post-block self-assessment included questions related to the frequency of the small-group tutoring participants and classroom tutoring participants applying during their clinical practice the knowledge taught during 3-month internal medicine additional courses. Frequency of course attendance was classified into always: 100%; frequently: 75–100%; often: 50–75%; occasionally: 25–50%; rarely: 0–25%. The committee notified the clerks that their names would remain attached to their responses until the pre-block and post-block responses were linked, at which time all identifying information would be removed.

### End-of 3-month internal medicine block formative “clinical excellence” OSCE

A formative 5-station OSCE was administered to all clerks in the classes 2013 and 2014 in order to evaluate their diagnostic and clinical reasoning skills. Taipei VGH has a national OSCE center that was established in 2003 with a well-organized OSCE committee and regular-qualified facility members. In the past 10 years plus, between 700 and 1000 multidisciplinary trainees have had their training efficiency certified at our OSCE center. Regularly, the committee of our OSCE center provides training courses to old and new raters, to standardized patients (SP), and to SP trainers (SPT) in order to satisfy the expanding needs of the rater/SP/SPT pool. During the 2013 and 2014 class year, the content of each post-Internal-Medicine “clinical excellence” OSCE station consisted of clinical scenario that had been certified by the clerkship education committee and had been developed by 15 faculty members. These 15 faculty members were invited from other medical centers and had well-certificated experience in OSCEs and medical education. In other words, these 15 faculty members who responsible for writing the OSCE clinical scenario are totally different from the faculty members who gave the small-group and classroom tutoring courses.

Each station lasts for 15 min and consists of 12 min of examination and 3 min immediate feedback by the raters. There is a single rest station (5 min) between the third and four OSCE station. Movement of the examinees from one station to another is controlled by a broadcast system located at the monitoring center. Our design consists of triplicate tracks, each containing five OSCE stations and the examinees and SP were randomly assigned to track. Almost all stations involved the rater who had developed that station with its checklist. In order to evaluate the 124–128 examinees of classes of 2013 and 2014 each year, the required four sessions with 15-OSCE stations lasting 6-h per day needed to be conducted over two consecutive weekend days.

At each OSCE station, the raters acted as passive evaluators to mark checklist and were instructed not to guide or prompt the examinees. The checklist at each station consisted of two parts, one for diagnostic skills and the other for clinical reasoning skills in order to provide two OSCE scores namely an OSCE_diag_ score and an OSCE_reason_ score, the results were collected by the clerkship OSCE committee for summation and final checking, then all scores were converted into 100 percentages.

### Statistical analysis

The internal consistency of the “clinical excellence” OSCE was good (Cronbach’s alpha = 0.72). The post-block OSCE_diag_ and OSCE_reason_ scores of the clerks who formed the small-group brainstorming tutoring course group, the classroom tutoring lecture group or the control group were compared using one-way ANOVA. Meanwhile, the between groups comparison of self-assessed degrees of “clinical excellence” were also analyzed using one-way ANOVA. Pearson’s correlation coefficient was used to measure the correlation between the overall OSCE_diag_ and OSCE_reason_ scores of small-group and classroom tutoring participants were also analyzed.

## Results

### Basal characteristics

In general, the mean age (21.2 ± 1.3 vs. 22.5 ± 0.7 year-old) and sex (% of male in total) ratio (35.9% vs. 35.7%) were not different between classes of 2013 and 2014 clerk. Additionally, the mean age (small-group: 22.5 ± 0.6, classroom: 21.8 ± 0.3, regular control: 20.5 ± 1.4 year-old) and sex (% of male in total) ratios (small-group: 34.9%, classroom: 35.3%, regular control: 37.5%) were similar among three groups of clerks [participants of small-group brainstorming tutoring course group (*n* = 84), the classroom tutoring lecture group (*n* = 86) and the control group (*n* = 82) who did not attend either of the above courses].

### Clerk’s self-assessment for “clinical excellence”

Both the small-group brainstorming tutoring group and the classroom tutoring group were found to have significantly higher degree of post-block self-assessed “clinical excellence” for all aspects of diagnostic and clinical reasoning skills compared to their pre-block self-assessment (Table 3). During the post-block self-assessment, there were significantly greater improvements in the clerk’s CXR, KUB, CBC + biochemistry diagnostic and clinical reasoning skills among small-group tutoring group than the classroom tutoring group. Basically, clerks forming part of the control group were more confidence of their diagnostic and clinical reasoning skills at the start of internal medicine block. However, this group’s post-block self-assessed degree of “clinical excellence” was not different from their pre-block self-assessed degree of “clinical excellence”. This lack of a significant change between the pre-block and post-block self-assessment of the regular controls might be the result of a lack of peer-pressure before the OSCE among the control group. Significantly, at end of the internal medicine block, the self-assessed degree of “clinical excellence” of small-group brainstorming tutoring group was higher than the other two groups, while that of the classroom tutoring group and the control group were similar.

**Table 3:** Comparison of the pre- and post-internal medicine block’s degree of self-assessed “clinical excellence” among three groups of class 2013 and 2014 clerks

	Small-group brainstorming course participants (*n* = 84)		Classroom tutoring course participants (*n* = 86)		Participants of control group (*n* = 82)	
	Pre-block	Post-block	Pre-block	Post-block	Pre-block	Post-block
I am good at basic electrocardiogram (EKG) diagnostic skills	3.2 ± 0.93*	4.7 ± 1.31^†^	3.06 ± 0.42*	4.1 ± 0.92^†^	4.1 ± 0.39	4.7 ± 0.77
I am good at basic chest X-ray (CXR) diagnostic skills	2.35 ± 1.09	4.4 ± 1.13^††^	2.19 ± 0.55	3.9 ± 0.87^#^,^†^	2.87 ± 0.89	3.2 ± 1.03^#^
I am good at basic abdominal plain film (KUB) diagnostic skills	2.84 ± 1.28	4.6 ± 1.16^†^	2.51 ± 1.04	3.5 ± 1.36^#^,^†^	2.94 ± 0.75	3.8 ± 0.64^#^
I am good at basic arterial blood gas diagnostic skills	2.95 ± 0.70*	4.8 ± 1.14^††^	3.04 ± 1.03*	4.0 ± 1.18^†^	3.56 ± 0.54	3.9 ± 0.99^#^
I am good at basic complete blood count and biochemistry diagnostic skills	2.65 ± 0.62	4.3 ± 0.78^†^	2.93 ± 0.82	3.6 ± 1.16^#^	3.2 ± 0.67	4.1 ± 1.3
I am good at image and laboratory-related clinical reasoning skills	2.23 ± 0.92*	4.5 ± 0.81^†^	2.78 ± 0.60	3.7 ± 1.15^#^,^†^	2.96 ± 0.91	3.4 ± 1.14^#^

Participants of control group: those did not attend either small-group or classroom tutoring courses**;** 5-point Likert scale (1 = fully agree; 5 = I fully disagree); The class 2013 and 2014 pre-block and post-block nonparametric, ordered and paired self-assessments were analyzed using a paired Wilcoxon test

*P < 0.05 or **P < 0.01 vs. regular controls; ^#^ *P* < 0.05 or ^##^ *P* < 0.01 vs. small-group team tutoring courses tutees; ^†^ *P* <0.05 or ^††^ *P* < 0.01 vs. pre-block assessment

### The performance of all clerks during the “clinical excellence” OSCEs

Significantly, the small-group tutoring group was found to have higher overall OSCE_diag_ and OSCE_reason_ scores than either classroom tutoring group or the control group (Table 4). However, the overall OSCE_diag_ and OSCE_reason_ scores were not significantly different between classroom tutoring group and control group. When the EKG, CXR, KUB, ABG, CBC + biochemistry OSCE stations were examined individually, the diagnostic skill (OSCE_diag_) scores of small-group tutoring group were higher than those of the classroom tutoring group. By way of contrast, only the clinical reasoning skill (OSCE_reason_) scores for the EKG and CBC + biochemistry OSCE stations of the small-group tutoring group were higher than those of classroom tutoring group. In other words, the OSCE_reason_ scores for the CXR and KUB OSCE stations of the small-group and classroom tutoring group were not significantly higher than those of control group whose members had not attend any of the additional tutoring courses. These differences might be associated with additional tutoring sessions for CXR, KUB and ABG clinical reasoning skills were provided by radiology faculty rather than internal medicine faculty. At the same time the OSCE_reason_ scores obtained at the ABG station, both those of the small-group tutoring group or the classroom tutoring group, were relatively lower than obtained at the other four OSCE stations. This probably reflects less efficient training by the general practicing faculty, who presented the basic ABG diagnostic and clinical reasoning session, compared to more advance content taught by the chest medicine or nephrology faculty.

**Table 4:** Comparison of the performance of formative “clinical excellence” OSCE of three groups of class 2013 and 2014 clerks

	Small-group brainstorming course participants (*n* = 84)		Classroom tutoring course participants (*n* = 86)		Participants of control group (*n* = 82)	
	OSCE_diag_ scores	OSCE_reason_ scores	OSCE_diag_ scores	OSCE_reason_ scores	OSCE_diag_ scores	OSCE_reason_ scores
Basic EKG diagnostic and clinical reasoning station	91.2 ± 8.31*	88.3 ± 3.9**	82.3 ± ^#^	71.69 ± 4.8^##^	81.0 ± 2.7^#^	70.5 ± 3.4^#^
Basic CXR diagnostic and clinical reasoning station	90.1 ± 6.92**	82.1 ± 2.5	78.2 ± ^##^	81.2 ± 7.9	73.4 ± 1.9	80.9 ± 7.7
Basic KUB diagnostic and clinical reasoning station	94.5 ± 3.21*	73.5 ± 1.4	83.7 ± ^#^	70.2 ± 6.8	84.5 ± 3.2^#^	69.8 ± 3.4
Basic ABG diagnostic and clinical reasoning station	86.7 ± 3.97*	77.98 ± 4.2	79.4 ± ^##^	74.5 ± 2.9	76.9 ± 2.2^#^	75.7 ± 4.8
Basic CBC and biochemistry diagnostic and clinical reasoning station	89.2 ± 1.59*	96.4 ± 5.3**	81.6 ± ^#^	89.8 ± 4.4^#^	82.9 ± 1.7	84.5 ± 3.7^#^
Overall OSCE scores	445.4 ± 6.45**	424.2 ± 11**	405.2 ± 9^#^	387.4 ± 16^#^	398.7 ± 21^#^	381.4 ± 29^#^

*P < 0.05 or **P < 0.01 vs. regular controls; ^#^ *P* < 0.05 or ^##^ *P* < 0.01 vs. small-group team tutoring courses tutees

### Relationship between the pre-/post-block self-assessments and formative OSCE

It is clear that the better training provided by the small-group tutoring courses was able to enhancing the clerk’s excellence in clinical care and this was able to be identified through a well-organized subjective OSCE evaluation and by self-assessment. During their clinical clerkship, a higher reported percentage of frequent (actually 75–100% of clinical work time) application of the taught diagnostic and clinical reasoning skills was found among the small-group tutoring group (55%) than among the classroom tutoring group (29%).

### Comparison of all clerk’s overall OSCE_diag_ and OSCE_reason_ scores

Among the classes of 2013 and 2014, 27% of the clerks (34/124) and 39% of the clerks (50/128), respectively, volunteered to attend the active small-group brainstorming tutoring course (Table 5). Furthermore, 30% of the clerks (37/124) and 39% of the clerks (49/128), respectively, volunteered to attend the didactic classroom tutoring course. The remaining clerks formed the control group and consisted of 43% of the clerks (53/124) and 22% of the clerks (29/128), respectively, and these individuals did not attend either the active small-group course or the didactic classroom tutoring course (Table 5). The reported reasons for the clerks not joining either group were a reluctance to let the additional course impinge on their after-work and weekend studying times.

**Table 5:** The distribution (%) of small-group and classroom tutoring participants in high (>400) and low (<400) OSCE_diag_ and OSCE_reason_ score’s groups among class 2013 and 2014 clerks whose with different levels of additional course’s attendance

	School’s year	Small-group tutoring	Classroom tutoring	Regular control
Attendance (%) of two additional tutoring courses or not attending additional course (regular control) among all clerks [*case No. (% among class 2013 or class 2014 clerks*)]	Class 2013	34/124(27%)	36/124(30%)	54/124(43%)
	Class 2014	50/128(39%)*	50/128(39%)	28/128(22%)
Participants with high (>400) post-Internal Medicine block’s OSCE_diag_ scores	Class 2013	21/34 (62%)	22/36(61%)	20/54(37%)
	Class 2014	36/50 (72%)*	31/50(62%)	11/28(39%)
Participants with low (<400) post-Internal Medicine block’s OSCE_diag_ scores	Class 2013	13/34(38%)	14/36(39%)	34/54(63%)
	Class 2014	14/50(28%)	19/50(38%)	17/28(61%)
Participants with high (>400) post-Internal Medicine block’s OSCE_reason_ scores	Class 2013	22/34(65%)	23/36(64%)	19/54(35%)
	Class 2014	37/50(74%)*	34/50(68%)	10/28(36%)
Participants with low(<400) post-Internal Medicine block’s OSCE_reason_ scores	Class 2013	12/34(35%)	13/36(36%)	35/54(65%)
	Class 2014	13/50(26%)	17/50(32%)	18/28(64%)

Data of OSCE scores were expressed as cases number (% among class 2013 and class 2014 clerks in the same groups)

*P < 0.05 vs. corresponding percentage of class 2013 clerks

**Fig. 1:**
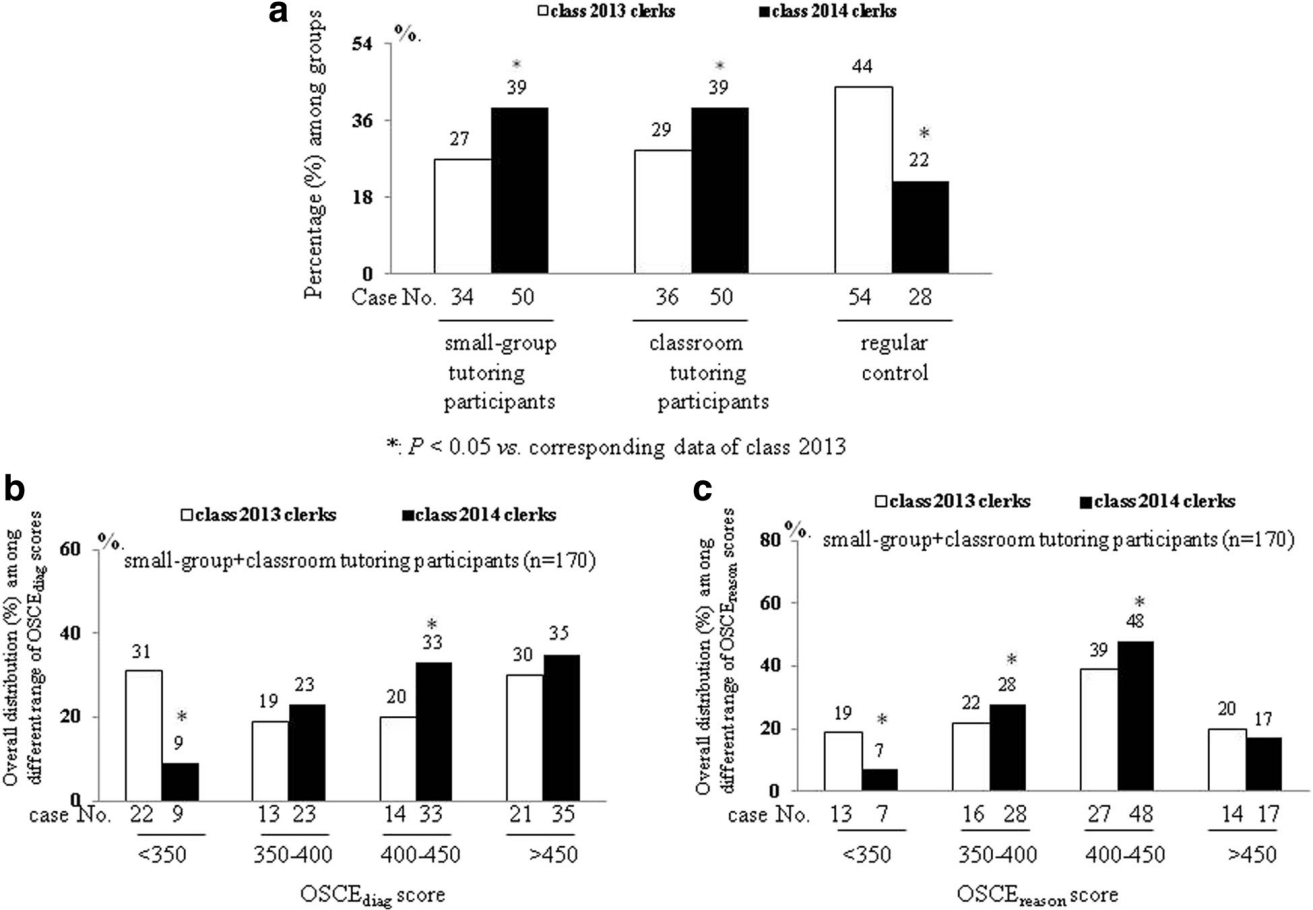
**a** Different attendance levels (percentage) of the class 2013 clerks and class 2014 clerks across the three group. **b** overall OSCE_diag_ and **c** OSCE_reason_ scores of the classes 2013 and 2014 clerks whose with different attendance levels in either small-group/classroom tutoring courses or absent of any course

In comparison with class 2013 clerks, the increasing participating trend of class 2014 clerks was observed both among the small-group brainstorming and classroom tutoring participants (Fig. 1a). Better attendance at one of these two additional tutoring courses resulted in the higher proportion of class 2014 clerks obtaining higher either overall OSCE_diag_ or OSCE_reason_ scores, especially in the range of 400–450, than class 2013 clerks (Fig. 1b-c).

**Fig. 2:**
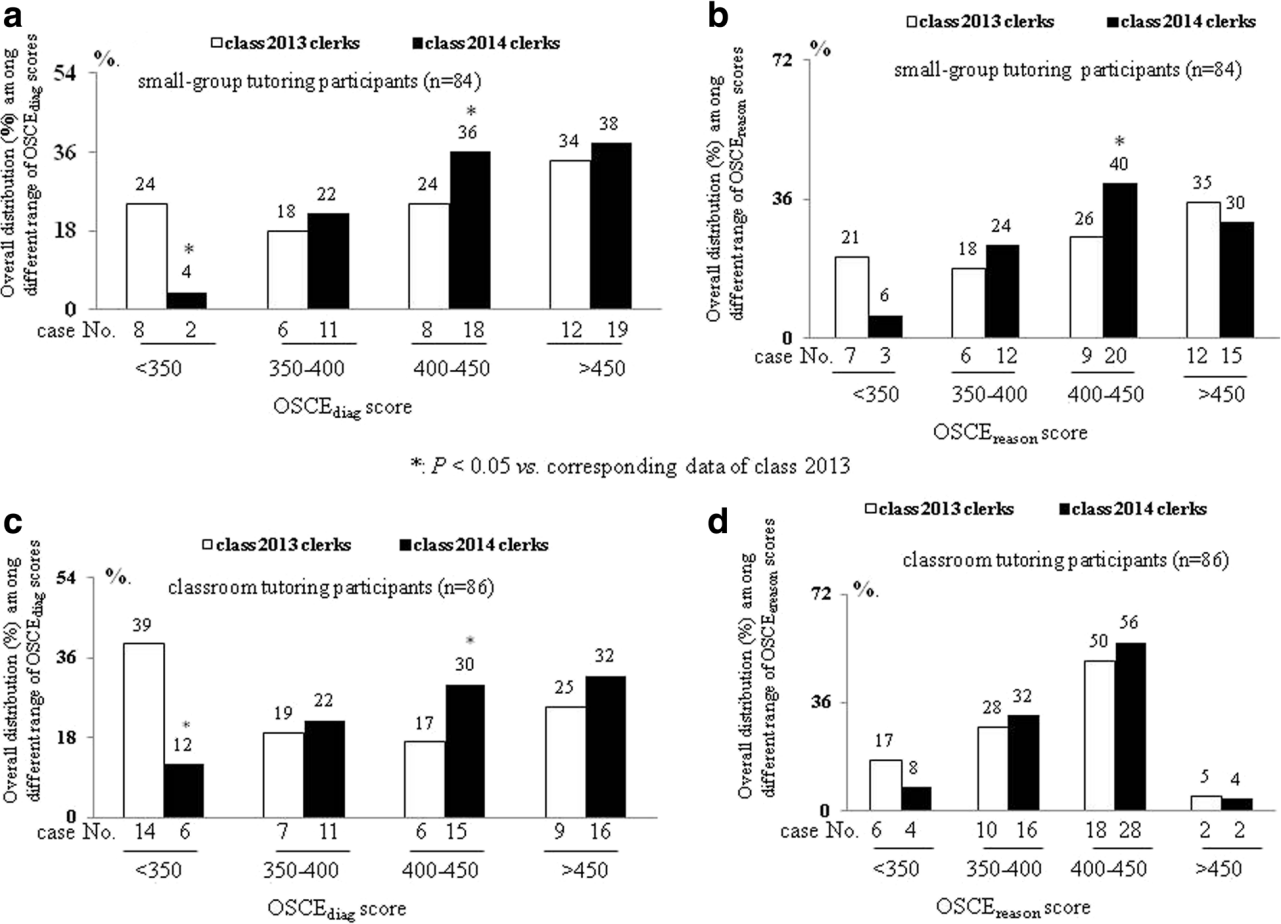
The distribution (%) of class 2013 and class 2014 small-group (**a**-**b**) and classroom tutoring (**c**-**d**) participants in different ranges [<350, 350–400, 400–450, >450] of OSCE_diag_ and OSCE_reason_ scores in their post-Internal Medicine block’s OSCE

In comparison with class 2013 clerks with low small-group tutoring attendance level, higher proportion [74 (36 + 38) % vs. 58 (24 + 34)%, Fig. 2a] of class 2014 clerks got high OSCE_diag_ scores (greater than 400) with increased (39% vs. 27%, Fig. 1a & Table 5) small-group tutoring attendance level. Especially, in the OSCE_diag_ or OSCE_reason_ scores ranged of 400–450, the significantly high percentage [OSCE_diag_: scores: 24% vs. 36%, Fig. 2a; OSCE_reason_ scores 26% vs. 40%, Fig. 2b] of class 2014 clerks was noted than class 2013 clerks in small-group tutoring group. However, above mentioned associated changes between attendance level and OSCE performance were not significant between class 2013 and class 2014 classroom tutoring participants (Figs. 1a and 2c-d, Table 5).

**Fig. 3:**
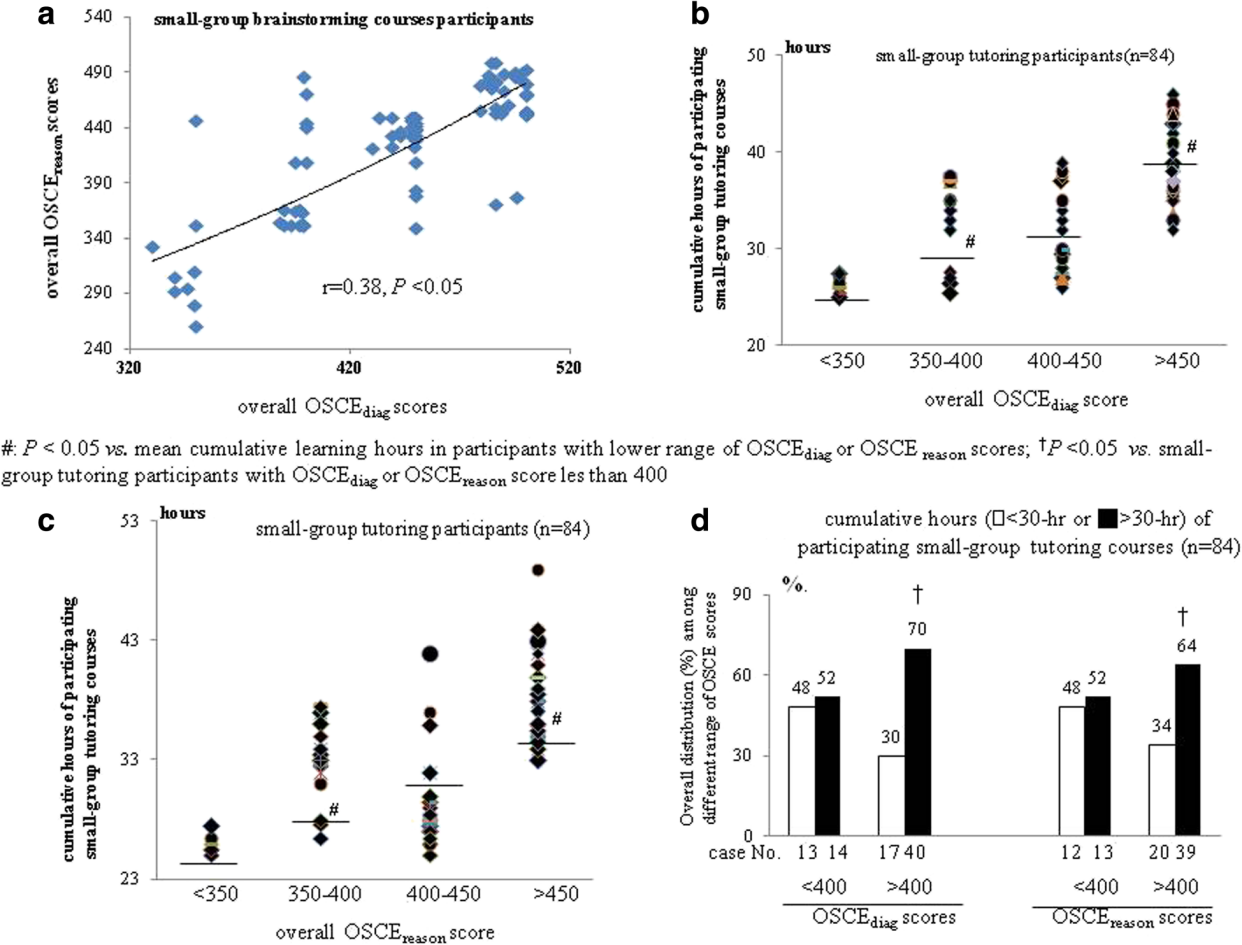
**a** Correlation between OSCE_diag_ and OSCE_reason_ scores of small-group tutoring participants; **b-c** The mean cumulating learning hours of small-group participants with stepwise increasing ranges of overall OSCE_diag_ and OSCE_reason_ scores (*horizontal black line in the dot plot* indicated mean); **d** Comparison of the proportion (%) of small-group tutoring that having high cumulative learning hours (>30-h) between high (>400) and low (<400) OSCE_diag_ and OSCE_reason_ score’s groups

Notably, a positive correlation (*r* = 0.38, *P* < 0.05) was noted between overall OSCE_diag_ and OSCE_reason_ scores of small-group tutoring participants (Fig. 3a). Significantly, mean of cumulative learning hour was sequential increased with the stepwise increasing ranges [<350, 350–400, 400–450, >450] of overall OSCE_diag_ and OSCE_reason_ scores in small-group tutoring participants (Fig. 3b-c). In small-group tutoring participants, the correlation between having OSCE_diag_/OSCE_reason_ scores greater than 400 and cumulative learning hours greater than 30 were observed among classroom tutoring participants (Fig. 3d).

**Fig. 4:**
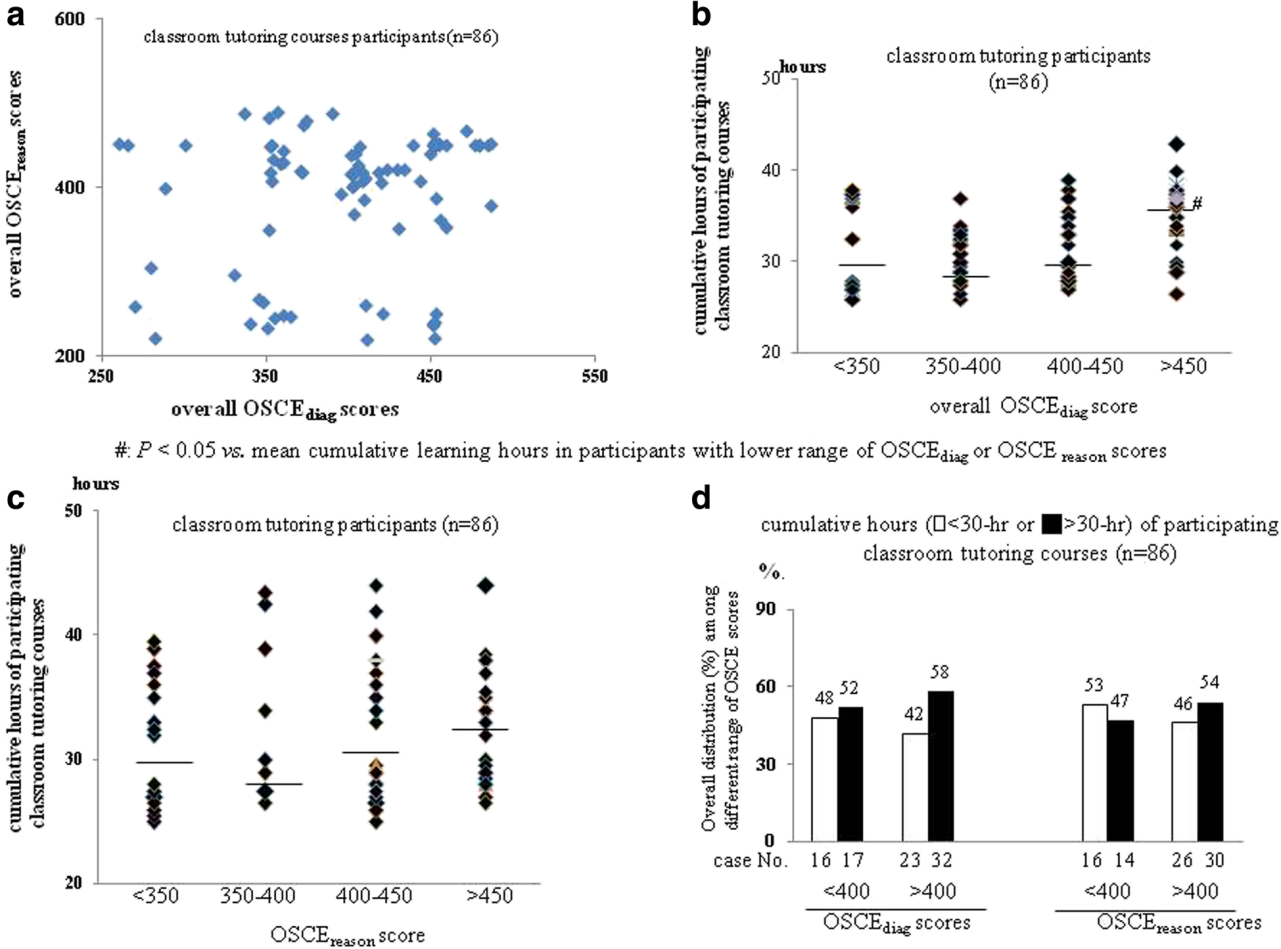
**a** Correlation between OSCE_diag_ and OSCE_reason_ scores of classroom tutoring participants; **b**-**c** The mean cumulating learning hours of classroom tutoring participants with stepwise increasing ranges of overall OSCE_diag_ and OSCE_reason_ scores (*horizontal black line in the dot plot* indicated mean); **d** Comparison of the proportion (%) of classroom tutoring participants that having high cumulative learning hours (>30-h) between high (>400) and low (<400) OSCE_diag_ and OSCE_reason_ score’s groups

Notably, there was no significant correlation between overall OSCE_diag_ and OSCE_reason_ scores of classroom tutoring participants (Fig. 4a). In classroom tutoring group, the significant incremental trend of mean of cumulating learning hour was not observed among participants with stepwise increasing ranges of overall OSCE_diag_ and OSCE_reason_ scores (Fig. 4b-c). In other words, the correlation between having OSCE_diag_/OSCE_reason_ scores greater than 400 and cumulative learning hours greater than 30 were not obvious among classroom tutoring participants (Fig. 4d).

## Discussion

The small-group brainstorming course and classroom tutoring courses that were part of our pilot clerkship “clinical excellence” programs offered nearly identical content exposure to all of the clerks who were voluntary participants. Notably, our programs were characterized by offering long exposure to these two diagnostic and clinical reasoning skills courses which encompassed the whole 3 months of the internal medicine block. Such a long exposure to these courses ought to help both the small-group brainstorming participants and classroom tutoring participants apply their newly acquired diagnostic and clinical reasoning skills in clinical practice. In reality, their immediate hands-on experiences will parallel the tutoring courses and this will enhancing their ability to actively build diagnostic and clinical reasoning skills, as well as allowing them to acquire an appropriate set of attitudes towards clinical works. Our voluntary participants responded positively regarding their experiences on these two additional tutoring courses and the broad spectrum of clinical scenario-based diagnostic and clinical reasoning training, as well as the dedication of their tutors.

Clerkship is the point in the career of medical students when they move from basic core academic training and begin to participate in clinical rotation. The fact that there are restriction on the duty hours of interns and residents in the current medical system means that it is important that clerks have competent diagnostic and clinical reasoning skills so that they are able to share properly in the responsibilities associated with patient care [9]. Each day, new clerks face a complex series of challenge in an intense clinical setting that involve a wide range of problems affecting difficult patients with serious illness and this is especially true for internal medicine wards. Interactive assessments revealed that medical students tend to overestimate their diagnostic skills [10]. In addition to technical skills, medical students entering clinical rotations have poor self-assessment of proficiency, low confidence, and high anxiety regarding diagnostic and clinical reasoning skills [8, 11–13]. Thus, dealing with complicated patients in a clinical situation requires the clerk to have placed emphasis on the building up of clerks’ diagnostic and clinical reasoning skills in order to promote excellence in clinical care among the clerks.

In particular, the small-group brainstorming courses provided participants with immediate feedback from other clerks and the member of faculty who was acting as the tutor; this allowed the group to develop appropriate diagnostic and clinical reasoning strategies. Previous studies had reported that learners respond well when given immediate small-group team-based feedback [14, 15]. In our study, it is reasonable to find that the satisfaction resulting from the immediate feedback, which is a character of small-group tutoring courses, was greater than the satisfaction resulting from the classroom-based didactic lectures.

Team-based small group learning is thought to improve a student’s perception of the value of working as a team. During our small-group brainstorming courses, the participants were given problems to stimulate discussion on the relevant topics; this allow them to pool their knowledge as they developed a consensus and ultimately this helped them to teach each other. The process, during which they struggled with a complex clinical scenario in a small-group setting, helped reinforce content objectives and brought learners to a higher level of understanding and cohesiveness [14–16]. In our study, the above mention advantages of small-group tutoring courses were translated into higher post-block self-assessment confidence, greater satisfaction and better performance in “clinical excellence” OSCE. Thus our small-group brainstorming tutoring courses impacted positively on the clerks’ diagnostic and clinical reasoning skills during their internal medicine block.

In the general surgical curriculum, implementation of faculty-led small group teaching instead of large group lecture-style dissemination improved the National Board of Medical Examiners (NBME) subject examinations performance [17]. In our study, the higher attendance levels increased the post-Internal Medicine block OSCE performance in small-group tutoring participants rather than classroom tutoring participants. It is suggested that participants did not get additional benefit from attending of classroom tutoring courses compared to regular control group. In other words, the context of didactic classroom tutoring can also obtain in their regular bedside teaching by clinical teachers.

In neurology clerkships, combined lecture-based learning with team-based learning produces better outcomes than either method alone [18]. Combining our and previous studies experience [14–18], the didactic classroom-based teachings need to be modified in terms of the setting, the content provided, the technology used and the training of faculty members who teach courses; these need to be implemented in a manner that matches the changing needs of young physicians whose are undertaking their medical education.

## Limitations

In our pilot study, all the clerks who participated in either the small-group brainstorming or the classroom-based didactic courses were volunteers who had a high motivation to learn; this may have resulted in some degree of some selective bias and this may have affected our results. This problem can be solving by randomly select clerks to each of the groups to avoid more studious and committed participants in small-group brainstorming group. Additionally, the large number of clinical teachers who supervised the clinical duties of the student during the participant’s clerkships necessarily varies in their diagnostic and clinical reasoning teaching skills and their level of experience in education. This factor can be modifying by balancing the training and choice of the faculty members across any future prospective randomized controlled trails. In addition to self-assessment, additional pre-Internal Medicine block OSCE might increase the reliability for ensuring that the possible bias from different “clinical excellence” levels among small-group, classroom tutoring and regular control group participants had been appropriately avoided in our study.

## Conclusions

In this pilot study, we introduced a small-group diagnostic and clinical reasoning brainstorming tutoring courses into the regular Internal Medicine clerkship and this was found to be highly rewarding by most of the voluntary participants found. They reported that they valued the clear learning objectives, their involvement in clinically integrated discussions that provided immediate feedback and confirmed that this complementary program ensure that they are excellence in clinical care.

## Additional files

Additional file 1: Table S1. Contents and results of the end-of-clerkship elf-assessed degree of excellence in clinical care of class 2012 clerks. (DOCX 15 kb)

Additional file 2: **Table S2** pre- and post-Internal Medicine block questionnaire for self-assessment of the degree of excellence in clinical care of class 2013 and 2014 clerks.

## Abbreviations

OSCEs: Objective subjective clinical examinations
