# Supplementary material for: Voluntary attendance of small-group brainstorming tutoring courses intensify new clerk’s “excellence in clinical care”: a pilot study
Source: BMC Med Educ. 2017 Jan 6;17:2. doi: 10.1186/s12909-016-0843-6 (PMC5217545; doi:10.1186/s12909-016-0843-6)
Supplement: Additional file 1: Table S1. — Contents and results of the end-of-clerkship elf-assessed degree of excellence in clinical care of class 2012 clerks. (DOCX 15 kb) [file 12909_2016_843_MOESM1_ESM.docx]

Additional file 1: Table S1 Contents and results of the end-of-clerkship elf-assessed degree of excellence in clinical care of class 2012 clerks

| I am good at | Degree of agreement (please give 1-5 point) |
| --- | --- |
| 1. Basic electrocardiogram (EKG) diagnostic skills | 2.66±0.64* |
| 1. Basic chest X-ray (CXR) diagnostic skills | 3.11±0.75* |
| 1. Basic abdominal plain film (KUB) diagnostic skills | 2.89±0.51* |
| 1. Basic arterial blood gas (ABG) diagnostic skills | 3.05±0.64* |
| 1. Basic complete blood count and biochemistry diagnostic skills | 2.35±1.02* |
| 1. image and laboratory-related clinical reasoning skills | 2.14±0.72* |
| 1. Write a progress note | 4.52±0.33 |
| 1. Identify legal and illegal practices | 4.7±0.42 |
| 1. Identify and protect private information | 4.61±0.6 |
| 1. Basic communicate skills | 3.9±1.1 |
| 1. Perform basic life support | 3.0±0.7* |
| 1. Start an intravenous injection (IV) | 2.98±0.8* |
| 1. Draw blood from a vein and artery (arterial blood gas) | 3.1±0.9* |
| 1. Place a nasogastric tube (NGT) | 3.0±1.1* |
| 1. Place a Foley catheter | 2.9±0.8* |

5-point Likert scale (1=fully agree; 5= I fully disagree); average agreement for 14 item is 3.3; *: the value that low than average is considering item needed to be improved.
